# Fatal Case of Cerebral Affection, with Certain Anomalous Symptoms Not Accounted for by the Appearances Found after Death

**Published:** 1826-07

**Authors:** 

**Affiliations:** the Hotel Dieu, Paris.


*Fatal Case of Cerebral Affection, with certain anomalous Symptoms not accounted for by the Appearances found after Death.* Treated by Dr. RECAMIER, at the HOTEL DIEU, Paris.

—— VERTOT, aged forty-two, journeyman; ill fifteen days altogether, during the last two of which he has been in the following state:—

November 22d, 1825.—Power of speech lost, being limited to some inarticulate monosyllables, when violently stimulated; intellectual functions considerably diminished, but still sufficient to induce him to show his tongue, and to make ineffectual attempts to speak. There is, however, no coma; and he opens his eyes from time to time, directing them with facility to different objects. The pupils are insensible and contracted; both the upper and lower eyelids are perfectly mobile, without unnatural contraction, and obedient to the will. The mouth is not drawn to one side; and the same is the case with respect to the tongue. The limbs are free, and their movements appear regulated. The neck is in a state of considerable rigidity, and allows the whole trunk to be raised by lifting up the head. The sensibility of the skin, examined on the extremities, the face, the thorax, and the abdomen, on either side, is in no degree impaired. The pulse is not frequent; the respiration is performed with freedom; the tongue is moist, and without coating; the bowels are confined, and there is retention of the urine. Lastly, the countenance is very little altered.

> Clyster, with a grain of tartarised antimony; bath at 27° of Reaumur; thirty leeches behind the ears; cupping along the vertebral column.

23d.—No change. Some remains of intelligence, but utter inability to pronounce a single word; nevertheless, the patient appears to hear. He is awake, and performs some voluntary movements, such as replacing the bed-clothes when he is uncovered. The general sensibility is the same; the neck and trunk continue stiff; the constipation and retention of urine remain; the pulse begins to augment in frequency.

> Bath; cupping along the vertebral column; castor oil; the catheter to be introduced.

24th.—Same state. Pulse augmenting in frequency; the pupils are still insensible, and perhaps less contracted.

this is the best mark of distinction between hysteria and the various inflammatory diseases which it mimics. During sleep, it generally falls to near its natural standard in hysterical affections.

The loss of speech is complete, although the patient still appears to hear; if this may be inferred from the different movements which he may be made to perform.

Bath; decoction of marshmallow for drink.

25th.—Tendency to sink down, (*affaissement;*) limbs less mobile, without however being paralysed; no diminution of the stiffness of the neck and trunk; pupils a little dilated; general sensibility impaired; aphonia continues; tongue dry and red; constipation; belly appears free from pain on pressure. Pulse ninety-four.

Tepid bath, with affusion; cupping along the vertebral column; potion of six ounces, with eight grains of tartarised antimony.

26th.—The state of the intellects is nearly the same, but he requires to be more strongly stimulated, in order to make him put out his tongue, or show other signs of intelligence. The neck and trunk remain fixed; the palsy of the bladder continues. The condition of the locomotive system of the limbs, and the general sensibility, has not varied since yesterday. The pupils are a little dilated; the pulse has diminished in frequency, being only eighty-eight in the minute. The patient appears to be more calm.

Potion with twelve grains of tartarised antimony; cupping glasses to be applied along the spine.

He died in the evening.

*Examination of the body.*—Head: The dura mater, the arachnoid, and the pia mater, exhibited *no unnatural appearance,* not the slightest trace of congestion or inflammation. The lateral ventricles scarcely contain an ounce of limpid lemon-coloured serosity; their serous membrane perfectly sound. The anterior part of the fornix very slightly softened, the change being sensible only to the finger, not at all so to the sight, and not occupying more than three or four lines at the most. The cerebral substance at the part was not at all injected, and without any change of colour. *The rest of the brain sound at the anterior lobe, as well as every other part: the same was the case with respect to the tuber annulare, the cerebellum, and the medulla oblongata.*

The spinal cord was natural, as well as its coverings, which exhibited no trace of congestion, unless in a very slight degree towards the extremity of the cauda equina.

The chest and abdomen were examined; but, as the details throw no light upon the case, we omit them.*

* Revue Medicale.

